# CALENDAR

**DOI:** 10.1054/bjoc.2000.2126

**Published:** 2001-10


					10-12 December 2001
Genes & Cancer 2001
UK Molecular Biology & Cancer Network Meeting XVII
University of Warwick, UK
Posters and Trade Exhibition
Transcriptional regulation, Tumour biology, Checkpoints,
New technologies
Further information from:
Dr Stefan Roberts, School of Biological Sciences, Manchester
University, Stopford Building, Oxford Road, Manchester M13
9PT, UK. Tel: + 44(0) 161 275 5214; Fax: + 44(0) 161 275 5600;
E-mail: stefan.roberts@man.ac.uk,www.icr.ac.uk/ukmbcn/info.htm
30 May-1 June 2002
The Second International Symposium on Target Volume
Definition in Radiation Oncology
Limburg/Lahn, Germany
The programme will include next year the following subjects:
digestive system tumours, thyroid cancer, gynecological tumours
and CNS tumours.
Further information from:
Assoc. Prof. Dr IC Kiricuta, Institute for Radiation Oncology,
St Vincenz-Hospital, Auf dem Schafsberg, 65549 Limburg/Lahn,
Germany. Tel: + 49 6431 292 4598; Fax: + 49 6431 292 4163;
E-mail: ic.chiricuta@st-vincenz.de
3-6 June 2002
Critical Issues in Tumor Microcirculation, Angiogenesis
and Metastasis: Biological Significance and Clinical
Relevance
Boston, Massachusetts, USA
A Continuing Education Course of Harvard Medical School and
Massachusetts General Hospital
Further information from:
http:// steele.mgh.harvard.edu
9-11 June 2002
UK Radiological Congress 2002
Birmingham, UK
Further information from:
UKRC Secretariat, The Conference Office, UKRC 2002, PO Box
2895, London, WIA5RS. Tel: +44(0)207 307 1410/1420;
Fax: +44(0)207 307 1414; E-mail: conference@ukrc.org.uk
CALENDAR
British Journal of Cancer (2001) 85(8), 1231
(c) 2001 Cancer Research Campaign
doi: 10.1054/ bjoc.2001.2126, available online at http://www.idealibrary.com on
1231
http://www.bjcancer.com

				

